# Phenylephrine versus norepinephrine for initial hemodynamic support of patients with septic shock: a randomized, controlled trial

**DOI:** 10.1186/cc7121

**Published:** 2008-11-18

**Authors:** Andrea Morelli, Christian Ertmer, Sebastian Rehberg, Matthias Lange, Alessandra Orecchioni, Amalia Laderchi, Alessandra Bachetoni, Mariadomenica D'Alessandro, Hugo Van Aken, Paolo Pietropaoli, Martin Westphal

**Affiliations:** 1 Department of Anesthesiology and Intensive Care, University of Rome, 'La Sapienza', Viale del Policlinico 155, Rome 00161, Italy; 2 Department of Anesthesiology and Intensive Care, University Hospital of Muenster, Albert-Schweitzer-Straße 33, Muenster 48149, Germany; 3 Laboratory of Clinical Pathology, Department of Surgery, University of Rome, 'La Sapienza', Viale del Policlinico 155, Rome 00161, Italy

## Abstract

**Introduction:**

Previous findings suggest that a delayed administration of phenylephrine replacing norepinephrine in septic shock patients causes a more pronounced hepatosplanchnic vasoconstriction as compared with norepinephrine. Nevertheless, a direct comparison between the two study drugs has not yet been performed. The aim of the present study was, therefore, to investigate the effects of a first-line therapy with either phenylephrine or norepinephrine on systemic and regional hemodynamics in patients with septic shock.

**Methods:**

We performed a prospective, randomized, controlled trial in a multidisciplinary intensive care unit in a university hospital. We enrolled septic shock patients (n = 32) with a mean arterial pressure below 65 mmHg despite adequate volume resuscitation. Patients were randomly allocated to treatment with either norepinephrine or phenylephrine infusion (n = 16 each) titrated to achieve a mean arterial pressure between 65 and 75 mmHg. Data from right heart catheterization, a thermodye dilution catheter, gastric tonometry, acid-base homeostasis, as well as creatinine clearance and cardiac troponin were obtained at baseline and after 12 hours. Differences within and between groups were analyzed using a two-way analysis of variance for repeated measurements with group and time as factors. Time-independent variables were compared with one-way analysis of variance.

**Results:**

No differences were found in any of the investigated parameters.

**Conclusions:**

The present study suggests there are no differences in terms of cardiopulmonary performance, global oxygen transport, and regional hemodynamics when phenylephrine was administered instead of norepinephrine in the initial hemodynamic support of septic shock.

**Trial registration:**

ClinicalTrial.gov NCT00639015

## Introduction

The current guidelines for the management of patients with septic shock recommend norepinephrine or dopamine as first-line agents to increase peripheral vascular resistance and to preserve organ perfusion following adequate volume therapy [1]. Moreover, the Surviving Sepsis Campaign recommends that phenylephrine should not be used as the initial vasopressor in septic shock [1], since phenylephrine may reduce splanchnic blood flow and oxygen delivery in septic shock patients [2,3]. Nevertheless, it is important to note that these recommendations are based on a limited number of studies that have evaluated the clinical use of phenylephrine in septic shock [2,4,5]. More importantly, a direct comparison between phenylephrine and norepinephrine in human septic shock has not yet been performed.

In contrast to norepinephrine that stimulates α_1 _and α_2 _receptors, and to a lower extent β_1 _and β_2 _receptors, phenylephrine is a selective α_1_-receptor agonist mainly constricting larger arterioles and having virtually no effects on terminal arterioles [6].

Krejci and colleagues recently compared the effects of norepinephrine and phenylephrine on microcirculatory blood flow in multiple abdominal organs in a porcine model of sepsis [7]. Whereas the norepinephrine-induced increase in perfusion pressure was associated with blood flow distribution away from the mesenteric circulation, phenylephrine did not impair the mesenterial blood flow distribution – suggesting possible beneficial properties of phenylephrine on hepatosplanchnic perfusion in septic shock.

In contrast, previous studies have reported that a delayed administration of phenylephrine replacing norepinephrine in a series of septic shock patients caused a more pronounced hepatosplanchnic vasoconstriction as compared with norepinephrine [2,8].

In the past few years, it has become evident that the efficacy of hemodynamic optimization by fluids and vasopressor agents critically depends on the urgency of therapy [1,9-11]. In this regard, it is conceivable that the negative effects of hepatosplanchnic perfusion noticed in response to phenylephrine administration [2,8] might have been related to a delayed treatment [11].

On this basis, we hypothesized that – compared with norepinephrine – early administration of phenylephrine does not worsen hepatosplanchnic perfusion during initial hemodynamic support of patients with septic shock. We therefore conducted a randomized, double-blind, controlled clinical trial to compare the effects of a first-line therapy with either phenylephrine or norepinephrine infusion on systemic and regional hemodynamics in patients with septic shock.

## Materials and methods

### Patients

After approval by the Local Institutional Ethics Committee, the study was performed in an 18-bed multidisciplinary intensive care unit (ICU) of the Department of Anesthesiology and Intensive Care of the University of Rome 'La Sapienza'. Informed consent was obtained from the patients' next of kin, as the patients were sedated and mechanically ventilated and thus were unable to give consent themselves. Enrollment of the patients started in December 2007 and ended in July 2008. This study has been registered as ClinicalTrial.gov NCT00639015. We enrolled patients who fulfilled the criteria of septic shock [1] presenting with a mean arterial pressure (MAP) below 65 mmHg despite appropriate volume resuscitation (pulmonary artery occlusion pressure (PAOP) = 12 to 18 mmHg and central venous pressure = 8 to 15 mmHg) [1].

Exclusion criteria were age <18 years, pronounced cardiac dysfunction (that is, cardiac index ≤ 2.2 l/min/m^2 ^in the presence of PAOP >18 mmHg), chronic renal failure, severe liver dysfunction (Child-Turcotte-Pugh grade C), significant valvular heart disease, present coronary artery disease, pregnancy, and present or suspected acute mesenteric ischemia.

All patients received mechanical ventilation using a volume-controlled mode with a plateau pressure maintained below 30 cmH_2_O [1]. All patients were appropriately analgo-sedated using sufentanil and midazolam.

### Measurements

Systemic hemodynamic monitoring of the patients (Vigilance^® ^II; Edwards Lifesciences, Irvine, CA, USA) involved a pulmonary artery catheter (7.5-F; Edwards Lifesciences) and a radial artery catheter (20 G; Arrow International Inc, Reading, PA, USA). The MAP, right atrial pressure, mean pulmonary arterial pressure, and PAOP were measured at end expiration. The heart rate was analyzed from a continuous recording of the electrocardiogram with ST segments monitored. The cardiac index was measured using the continuous thermodilution technique (Vigilance^® ^II; Edwards Lifesciences). The stroke volume index, systemic vascular resistance index, pulmonary vascular resistance index, left ventricular stroke work index, right ventricular stroke work index, oxygen delivery index, oxygen consumption index, and oxygen extraction ratio were calculated using standard formulae. Arterial and mixed-venous blood samples were taken for measuring oxygen tensions and saturations, as well as carbon dioxide tensions, standard bicarbonate, arterial base excess, pH, and arterial lactate. In addition, arterial blood samples were drawn for the determination of cardiac troponin I and creatinine concentrations.

Regional hemodynamic monitoring of the patients was performed with a 4-F oximetry thermodye dilution catheter (PV2024L; Pulsion Medical Systems AG, Munich, Germany) inserted through the femoral artery for the determination of the plasma disappearance rate of indocyanine green (PDR) and the blood clearance of indocyanine green related to body surface area (CBI). Moreover, an air tonometer (Tonocap; Datex-Ohmeda, Helsinki, Finland) was inserted via the nasogastric route for gastric mucosal carbon dioxide tension measurement.

The PDR and CBI were determined with the thermodye dilution method as assessed by the Cold Z-021 (Pulsion Medical Systems AG) using an established protocol [12,13]. Every value was calculated as the mean of three measurements, each consisting of a bolus of 0.3 mg/kg indocyanine green at 2 mg/ml (Pulsion Medical Systems AG) in ice-cold 5% glucose solution injected into the right atrium. In addition, the gradient between gastric mucosal and arterial pCO_2 _was calculated, which has been shown to be more appropriate for the detection of regional ischemia than for the calculation of mucosal pH [14,15]. Urine samples were collected to assess urinary output and creatinine clearance in the laboratory setting.

### Study design

Patients who met the entry criteria were randomized using a computer-based procedure, to receive either an infusion of phenylephrine or norepinephrine in a double-blinded fashion for 12 hours. The two study drugs were titrated to maintain a MAP between 65 and 75 mmHg. Serial fluid challenges were performed to maintain the central venous pressure at 8 to 15 mmHg and the PAOP between 12 and 18 mmHg during the 12-hour intervention period [1]. Packed red blood cells were transfused when hemoglobin concentrations decreased below 8 g/dl. If the mixed-venous oxygen saturation was <65% despite appropriate arterial oxygenation (arterial oxygen saturation ≥ 95%) and hemoglobin concentrations ≥ 8 g/dl, dobutamine was administered (with a maximum dose of 20 μg/kg/min) to achieve mixed-venous oxygen saturation values ≥ 65% [1]. Systemic, pulmonary and regional hemodynamic measurements, laboratory variables, and blood gases were determined at baseline and 12 hours after randomization. Creatinine clearance was determined over a period of 12 hours.

At the end of the 12-hour study period, study drugs were gradually reduced and switched to open-labeled norepinephrine. If necessary, dobutamine was given according to the study protocol mentioned above.

### Statistical analyses

The main endpoint of the present study was the modifications of the PDR and CBI after phenylephrine administration as compared with the norepinephrine group. To detect a 30% difference in one of the measured variables (that is, PDR and CBI) with an expected standard deviation of 30%, a test power of 80% and an α-error probability of *P *< 0.05, a sample size of 16 subjects per group was required [16]. Data are expressed as the mean ± standard deviation, if not otherwise specified. Sigma Stat 3.10 software (SPSS, Chicago, IL, USA) was used for statistical analysis.

After confirming the normal distribution of all variables (Kolmogorov-Smirnov test), differences within and between groups were analyzed using a two-way analysis of variance for repeated measurements with group and time as factors. Time-independent variables were compared with one-way analysis of variance. In the case of significant group differences over time, appropriate *post hoc *comparisons (Student-Newman-Keuls test) were performed. Categorical data were compared using the chi-square test. For all tests, an α-error probability of *P *< 0.05 was considered statistically significant.

## Results

### Patients

**Figure 1:**
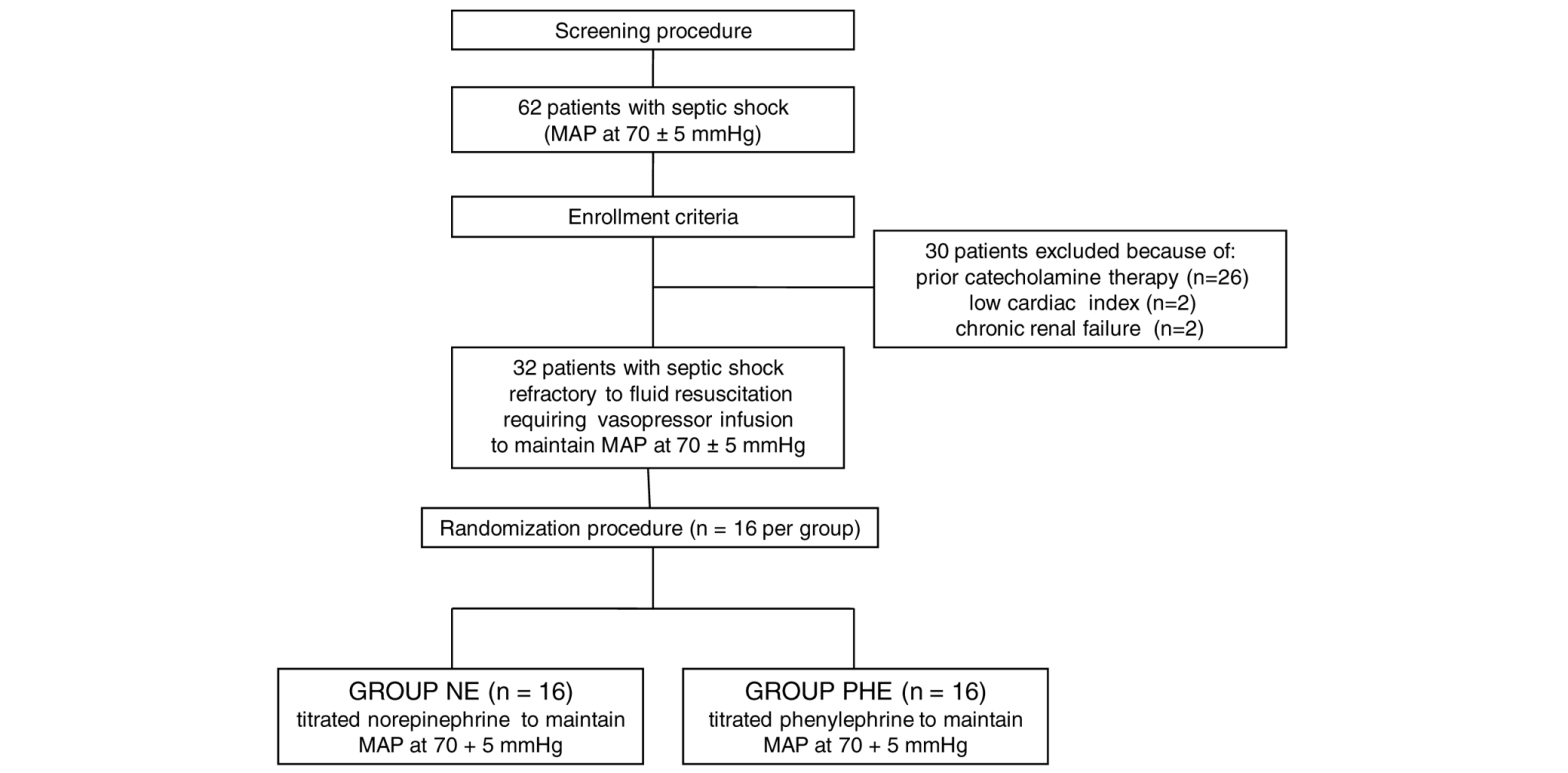
**Study design**. MAP, mean arterial pressure; NE, norepinephrine; PHE, phenylephrine.

After screening 62 patients with septic shock who met the inclusion criteria of the study, 30 patients had to be excluded due to prior catecholamine therapy (n = 26), inappropriately low cardiac output (n = 2), or chronic renal failure (n = 2). Finally, 32 consecutive patients were enrolled in the study and equally randomized into the two study groups (n = 16 per group) (Figure 1).

### Demographic data

Baseline characteristics including age, gender, body weight, origin of septic shock, and Simplified Acute Physiology Score II are presented in Table 1. There were no significant differences in baseline characteristics between groups, except for a higher body weight in the norepinephrine group. No differences were found between the phenylephrine and norepinephrine groups in the mean time elapsed from ICU admission to the need for vasopressor support (39 ± 35 hours versus 37 ± 38 hours, *P *= 0.282). In this regard, vasopressor administrations were initiated as soon as the inclusion criteria were met (with no time delay).

**Table 1:** Baseline characteristics of study patients

	Phenylephrine (n = 16)	Norepinephrine (n = 16)	*P *value
Age (years)	70 (53 to 74)	70 (59 to 74)	0.850
Gender (percentage male)	75	56	0.457
Simplified Acute Physiology Score II	57 ± 8	55 ± 7	0.434
Cause of septic shock	Pneumonia (n = 7), peritonitis (n = 8), meningitis (n = 1)	Pneumonia (n = 8), peritonitis (n = 8), meningitis (n = 0)	0.587
Mortality (n (%))	10/16 (63%)	9/16 (56%)	1.000
Intensive care unit length of stay (days)	16 (7 to 25)	16 (10 to 24)	0.597

Data presented as median (25% to 75% range) or mean ± standard deviation unless otherwise indicated.

### Study drug requirements and systemic hemodynamics

**Figure 2:**
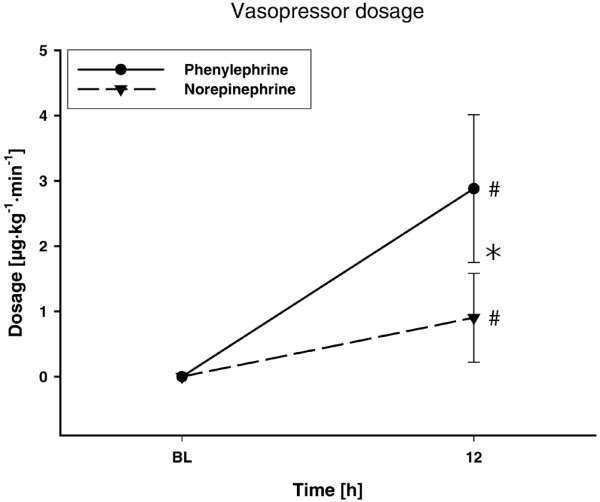
**Study drug requirements of study patients**. Vasopressor dosage throughout the study. ^#^*P *< 0.05 versus baseline (BL) (significant time effect). *P < 0.05, phenylephrine versus norepinephrine.

**Figure 3:**
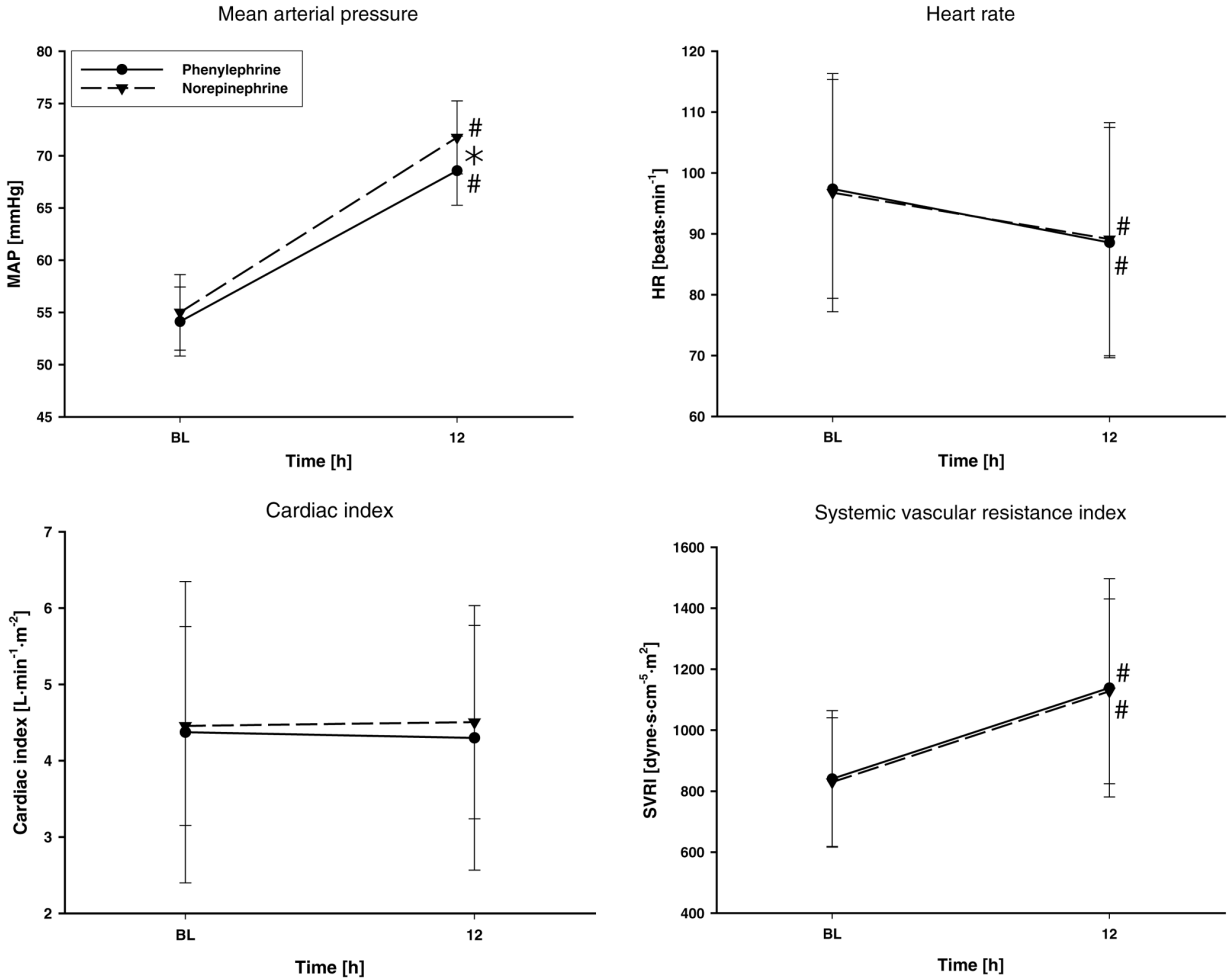
**Systemic hemodynamics of study patients**. Patients' mean arterial pressure (MAP), heart rate (HR), cardiac index, and systemic vascular resistance index (SVRI) throughout the study. ^#^*P *< 0.05 versus baseline (BL) (significant time effect). *P < 0.05, norepinephrine versus phenylephrine.

The amount of fluids infused during the study period in the phenylephrine and norepinephrine groups was similar (2,554 ± 1,140 ml versus 2,431 ± 1,010 ml, *P *= 0.751). Phenylephrine dosages were higher than those for norepinephrine 12 hours after randomization (*P *< 0.001) (Figure 2). The goal MAP of 65 to 75 mmHg was reached in all subjects. Twelve hours after randomization, the MAP was significantly higher in the norepinephrine group as compared with patients treated with phenylephrine (*P *= 0.011) (Figure 3). This difference remained, however, within the predefined threshold MAP of 65 to 75 mmHg. There were no significant differences between groups in any other variable of systemic hemodynamics (Figure 3 and Table 2).

**Table 2:** Hemodynamic variables of study patients

	Baseline	12 hours	*P *value
Pulmonary artery occlusion pressure (mmHg)			1.000
Phenylephrine	15 ± 2	17 ± 3*	
Norepinephrine	15 ± 2	17 ± 3*	
Right atrial pressure (mmHg)			0.902
Phenylephrine	13 ± 3	15 ± 3*	
Norepinephrine	13 ± 3	14 ± 3*	
Mean pulmonary arterial pressure (mmHg)			0.521
Phenylephrine	28 ± 9	33 ± 11*	
Norepinephrine	27 ± 5	30 ± 4*	
Pulmonary vascular resistance index (dyne·s/cm^5^/m^2^)			0.330
Phenylephrine	293 ± 253	348 ± 296*	
Norepinephrine	235 ± 103	264 ± 105	
Right ventricular stroke work index (g/m^2^/beat)			0.564
Phenylephrine	9 ± 5	12 ± 7*	
Norepinephrine	8 ± 4	11 ± 4*	
Left ventricular stroke work index (g/m^2^/beat)			0.721
Phenylephrine	25 ± 11	35 ± 14*	
Norepinephrine	25 ± 8	37 ± 9*	
Stroke volume index (g/m^2^/beat)			0.963
Phenylephrine	45 ± 18	49 ± 19	
Norepinephrine	46 ± 13	50 ± 11	
Cardiac troponin I (ng/ml)			0.854
Phenylephrine	1.0 ± 0.9	1.1 ± 0.9	
Norepinephrine	0.9 ± 0.9	1.1 ± 0.8	

*P < 0.05 versus baseline (significant time effect).

Whereas the heart rate significantly decreased in both study groups (*P *= 0.009 and *P *= 0.022 for phenylephrine and norepinephrine treatment versus baseline, respectively), the systemic vascular resistance index and the left ventricular stroke work index both increased as compared with baseline (each *P *< 0.001). The pulmonary vascular resistance index increased with time only in the phenylephrine group (*P *= 0.02 versus baseline). Six patients in the norepinephrine group as well as eight patients in the phenylephrine group received dobutamine during the study period (chi-square test: not significant and *P *= 0.722, respectively). The dobutamine requirements, however, were similar between the two groups (15 ± 5 μg/kg/min versus 14 ± 6 μg/kg/min, *P *= 0.35). The incidence of new-onset tachyarrhythmias was 2/16 in the phenylephrine and 1/16 in the norepinephrine group (chi-square test: not significant and *P *= 1.0, respectively).

### Regional hemodynamics, acid-base homeostasis, and oxygen transport variables

**Figure 4:**
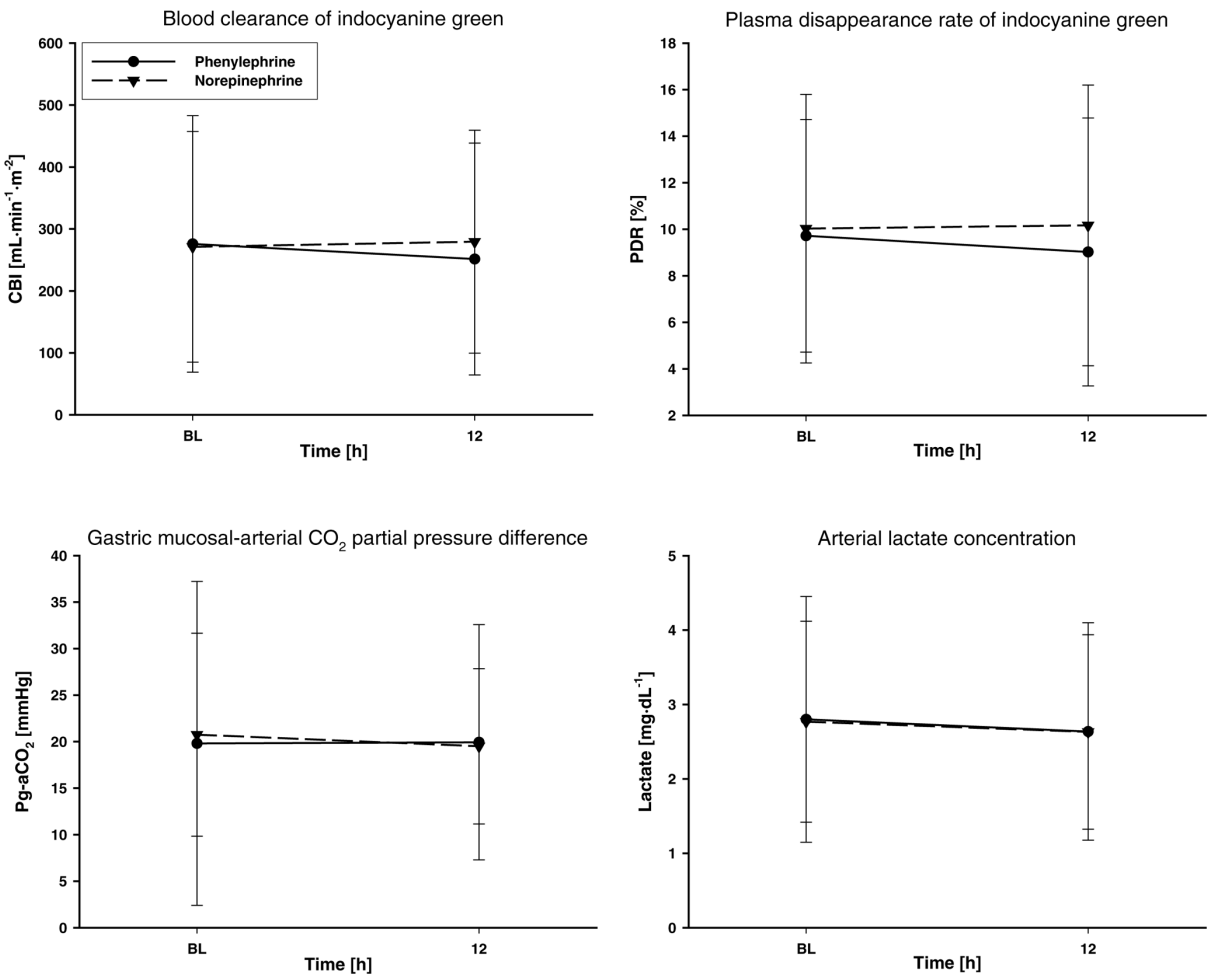
**Regional hemodynamics of study patients**. Patients' blood clearance of indocyanine green related to body surface area (CBI), plasma disappearance rate of indocyanine green (PDR), gradient between gastric mucosal and arterial pCO_2 _(p_g-a_CO_2_), and arterial lactate concentration throughout the study. BL, baseline.

There were no significant overall differences between groups in any variable of regional hemodynamics, acid-base homeostasis, or oxygen transport (Figure 4 and Table 3).

**Table 3:** Global oxygen transport variables and acid-base balance of study patients

	Baseline	12 hours	*P *value
Oxygen delivery index (ml/min/m^2^)			0.951
Phenylephrine	500 ± 205	498 ± 177	
Norepinephrine	499 ± 139	506 ± 140	
Oxygen consumption index (ml/min/m^2^)			0.568
Phenylephrine	164 ± 48	150 ± 41	
Norepinephrine	173 ± 53	161 ± 58	
Oxygen extraction ratio (%)			0.816
Phenylephrine	34 ± 9	32 ± 8	
Norepinephrine	36 ± 11	32 ± 10*	
Hemoglobin (g/dl)			0.699
Phenylephrine	8.4 ± 0.9	8.4 ± 1.1	
Norepinephrine	8.3 ± 0.7	8.4 ± 0.6	
pHa (-log_10_c(H^+^))			0.435
Phenylephrine	7.37 ± 0.07	7.37 ± 0.08	
Norepinephrine	7.35 ± 0.09	7.34 ± 0.08	
Arterial base excess (mmol/l)			0.228
Phenylephrine	-0.2 ± 5.8	0.2 ± 6.3	
Norepinephrine	-2.4 ± 6.6	-3.0 ± 6.4	
Arterial carbon dioxide partial pressure (mmHg)			0.346
Phenylephrine	44 ± 10	44 ± 10	
Norepinephrine	41 ± 6	41 ± 6	
Arterial oxygen partial pressure (mmHg)			0.963
Phenylephrine	128 ± 36	119 ± 31	
Norepinephrine	121 ± 24	120 ± 26	
Arterial oxygen saturation (%)			0.912
Phenylephrine	98 ± 2	98 ± 2	
Norepinephrine	98 ± 1	98 ± 2	
Mixed venous oxygen saturation (%)			0.431
Phenylephrine	66 ± 9	67 ± 9	
Norepinephrine	64 ± 11	67 ± 10	
P_a_O_2_/FiO_2_			0.971
Phenylephrine	229 ± 103	208 ± 89	
Norepinephrine	222 ± 75	218 ± 71	

P_a_O_2_/FiO_2_, ratio of arterial oxygen partial pressure and inspiratory oxygen fraction (Horovitz index). *P < 0.05 versus baseline (significant time effect).

### Variables of organ function and injury

**Figure 5:**
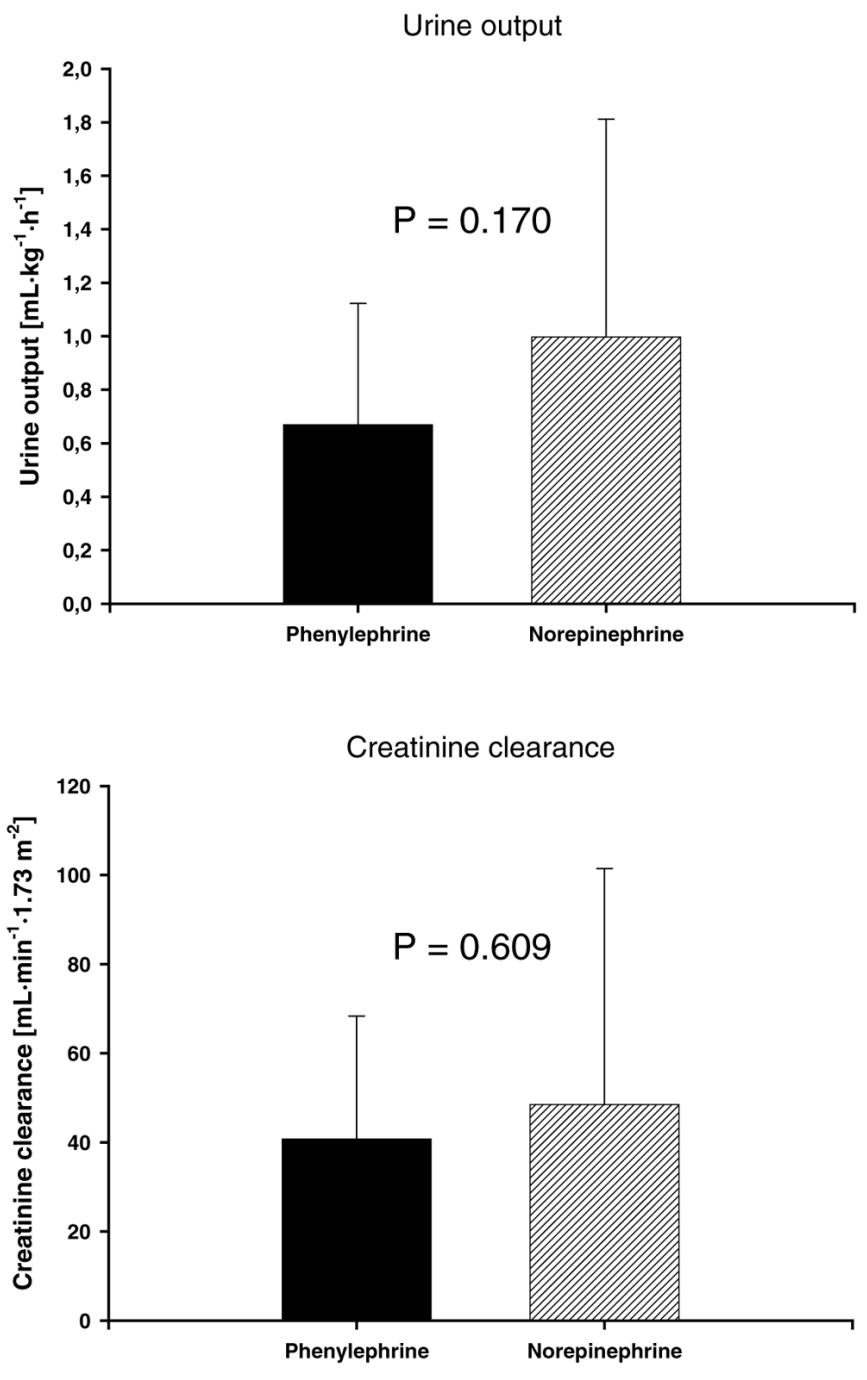
**Variables of renal function**. Urine output and creatinine clearance in the two treated patient groups.

Urine output and creatinine clearance were similar between groups throughout the 12-hour interventional period (*P *= 0.170 and *P *= 0.609, respectively) (Figure 5). Likewise, troponin I plasma concentrations were comparable between groups (Table 2).

### Length of ICU stay and outcome

The length of ICU stay and the ICU mortality were similar between groups (Table 1).

## Discussion

The major findings of the present study are that, when administered as a first-line vasopressor agent in septic shock patients, phenylephrine did not worsen hepatosplanchnic perfusion as compared with norepinephrine, had similar effects as norepinephrine on cardiopulmonary performance and global oxygen transport, and was less effective than norepinephrine to counteract sepsis-related arterial hypotension as reflected by the higher dosages required to achieve the same goal MAP.

Phenylephrine increases systemic vascular resistance by selectively stimulating α_1 _adrenoceptors without a compensatory increase in myocardial contractility, and thus in cardiac output [6]. From a hemodynamic point of view, it might be argued that, in volume-resuscitated patients, norepinephrine may potentially be advantageous over phenylephrine, since it simultaneously stimulates α_1_, β_1 _and β_2 _receptors, thereby counteracting arterial hypotension by increasing systemic vascular resistance and possibly myocardial inotropy [6]. On the other hand, phenylephrine could be preferable over norepinephrine, since β_1_-receptor stimulation may increase the heart rate and myocardial oxygen demand. In this regard, a previous study reported that prolonged tachycardia may increase the incidence of major cardiac events in critically ill patients [17].

In the present study we did not find any differences between groups treated with either norepinephrine or phenylephrine in terms of systemic hemodynamics. We recently reported that, in a series of septic shock patients, the systemic hemodynamics and global oxygen transport remained unchanged after replacing norepinephrine with phenylephrine except for a significant decrease in heart rate [8]. The different severity of the cardiovascular dysfunction among the studied patients, however, could have affected the results of the latter study [8]. In addition, the investigated patients were already treated with high norepinephrine dosages (0.8 ± 0.7 μg/kg/min) at study entry. It is therefore conceivable that – different from delayed treatment [8] – early administration of phenylephrine in the hypotensive patients enrolled in the present study could have played a pivotal role in this regard.

Nevertheless, at the end of the study period, phenylephrine dosages were higher than (that is, 220%) those for norepinephrine to maintain the predefined threshold MAP. Although a comparative dose-finding study in human septic shock has not yet been performed, our observation suggests that phenylephrine may be less effective as compared with norepinephrine to counteract arterial hypotension when high dosages of catecholamines are required.

Clinical evidence indicates that infusion of norepinephrine doses ranging from 0.01 to 3 μg/kg/min neither worsen splanchnic perfusion nor compromise organ function in the presence of septic shock [3,18-24].

Whereas only few clinical studies including a small number of patients have been performed on phenylephrine in septic shock [2,4,5,8], several studies have evaluated the impact of phenylephrine on splanchnic perfusion in experimental septic shock. In this regard, Breslow and colleagues reported no differences between phenylephrine (5.9 ± 2.7 μg/kg/min) and norepinephrine (3.0 ± 1.6 μg/kg/min) in terms of the splanchnic oxygen supply [25]. These findings were confirmed by Schwarz and colleagues, who reported that – despite major differences in systemic hemodynamics – progressively increasing phenylephrine from 0.1 to 10 μg/kg/min did not decrease jejunal tissue oxygen supply as compared with norepinephrine (from 0.01 to 2 μg/kg/min) [18]. In endotoxemic dogs, Zhang and colleagues likewise demonstrated that 1 μg/kg/min phenylephrine influenced neither hepatosplanchnic blood flow nor global and liver oxygen extraction capabilities [26]. Krejci and colleagues reported recently that norepinephrine in doses of 0.7 ± 0.3 μg/kg/min distributes blood flow away from the splanchnic circulation (for example, small intestine) to other regions of the body by the β-adrenergic stimulation [7]. Importantly, whereas norepinephrine reduced blood flow in both the jejunal mucosa and in the jejunal muscularis, phenylephrine at doses of 3.1 ± 1.0 μg/kg/min did not affect blood flow in the jejunal mucosa and even increased blood flow in the jejunal muscularis. It is therefore conceivable that an α_1_-receptor agonist such as phenylephrine -due to the lack of the β-adrenergic stimulation – may be beneficial in septic shock, because it increases blood pressure without causing negative effects on tissue blood flow.

In the clinical setting, Reinelt and colleagues reported that hepatosplanchnic oxygen delivery and blood flow decreased in six septic shock patients when norepinephrine was gradually replaced by phenylephrine at identical levels of MAP and cardiac index [2]. Our research group reported recently that, in a series of 15 septic shock patients, whereas phenylephrine did not impair gastrointestinal mucosal perfusion as measured by the gradient between gastric mucosal and arterial pCO_2_, it decreased hepatosplanchnic perfusion as indicated by a decrease in the PDR and CBI associated with a slight increase in arterial lactate concentration [8]. The latter study, however, was designed as a cross-over study replacing norepinephrine infusion with phenylephrine and then once again replacing with norepinephrine after 8 hours. Importantly, the patients involved were already treated with high norepinephrine dosages at study entry.

In the present study, phenylephrine administration did not negatively affect gastrointestinal perfusion (that is, the gradient between gastric mucosal and arterial pCO_2_) when compared with norepinephrine as first-line therapy in septic shock patients. The absence of detrimental splanchnic hemodynamic effects of phenylephrine during the observation period is further confirmed by the lack of overall differences between groups in terms of the PDR, CBI, acid-base homeostasis, as well as arterial lactate concentrations.

There are several reasons helping to explain the discrepancies between studies. First, in the studies of Reinelt and colleagues and of Morelli and colleagues, the MAP at baseline was 65 to 75 mm Hg [2,8], whereas it was considerably lower in the present study. Second, the mean time elapsed from meeting the criteria for study entry to infusion of phenylephrine was about 32 hours in the cited studies [2,8]. By contrast, in the present study, a different hemodynamic condition at baseline (that is, arterial hypotension) and, more importantly, the administration of phenylephrine at the time of shock onset could have played a pivotal role in this regard [12,27].

The effects of phenylephrine on renal function have not yet been fully elucidated. We recently reported that delayed administration of phenylephrine replacing norepinephrine in a series of septic shock patients negatively affected renal function, as indicated by a decrease in creatinine clearance compared with norepinephrine administration [8]. In the present study, we noticed no differences between the two study drugs in terms of urine output or creatinine clearance. The number of patients who required renal replacement therapy at the end of the 12-hour study period, however, although not statistically significant, tended to be higher in the phenylephrine group (7 patients versus 2 patients, *P *= 0.133). Although speculative, this finding supports the notion that mixed α-adrenergic and β-adrenergic agents when given to increase or maintain the MAP may better preserve renal blood flow as compared with sole α-agonists [28-31]. Nevertheless, the implication of this finding for the course of the disease remains uncertain and should be clarified in future studies.

The present study has some limitations that we would like to acknowledge. First, direct measurements of regional and local splanchnic blood flow in septic shock patients are invasive and require special skills and instruments that are not readily available at the bedside. In the present study, therefore, hepatosplanchnic perfusion was assessed using the PDR, CBI, and gastric tonometry as surrogates of hepatosplanchnic perfusion and function. Second, as phenylephrine was administered as a first-line vasopressor agent in the present study, for safety reasons we investigated only a small number of septic shock patients to evaluate the effects on cardiopulmonary and regional hemodynamics over a relative brief period (that is, 12-hour intervention period). We therefore cannot rule out the possibility of adverse metabolic alterations or worsening of hepatosplanchnic perfusion in response to administration of phenylephrine for a prolonged period. Third, even though it was possible to define the exact time when the enrolled patients required vasopressor support during the ICU stay, we cannot exclude differences in the time of onset of sepsis before ICU admission. Finally, since the present study was powered to demonstrate a 30% difference in the PDR and CBI, smaller differences, even though of scarce clinical implications, cannot be excluded by the present data. This question can only be answered by studies investigating a larger sample size.

## Conclusion

This is the first prospective, randomized, controlled study comparing systemic and regional hemodynamic effects of phenylephrine and norepinephrine infusion in the early phase of septic shock. Our results suggest that phenylephrine – when administered as a first-line vasopressor agent in septic shock – is effective in increasing the MAP without compromising gastrointestinal and hepatosplanchnic perfusion as compared with norepinephrine.

## Key messages

• There are no differences between norepinephrine and phenylephrine in terms of systemic hemodynamics when they are administered as a first-line vasopressor agent in septic shock.

• Phenylephrine is less effective than norepinephrine to counteract sepsis-related arterial hypotension.

• Phenylephrine does not impair gastrointestinal mucosal perfusion.

• Delayed administration of phenylephrine in septic shock patients causes a more pronounced hepatosplanchnic vasoconstriction as compared with norepinephrine.

• Phenylephrine – when administered as a first-line vasopressor agent in septic shock – is effective for increasing the MAP without compromising gastrointestinal and hepatosplanchnic perfusion, as compared with norepinephrine administration.

## Abbreviations

CBI: blood clearance of indocyanine green related to body surface area; MAP: mean arterial pressure; PAOP: pulmonary arterial occlusion pressure; pCO_2_: carbon dioxide partial pressure; PDR: plasma disappearance rate of indocyanine green.

## Competing interests

The authors declare that they have no competing interests.

## Authors' contributions

AM and MW conceived of the study, were responsible for its design and coordination, and helped to draft the manuscript. CE, ML, SR, and HVA participated in the design of the study, performed the statistical analysis, and helped to draft the manuscript. AO and AL participated in the study design and helped to draft the manuscript. AB and MD participated in the study design, performed laboratory measurements, and helped to draft the manuscript. PP participated in the study design and coordination, helped to draft the manuscript, and obtained funding. All authors read and approved the final manuscript.
